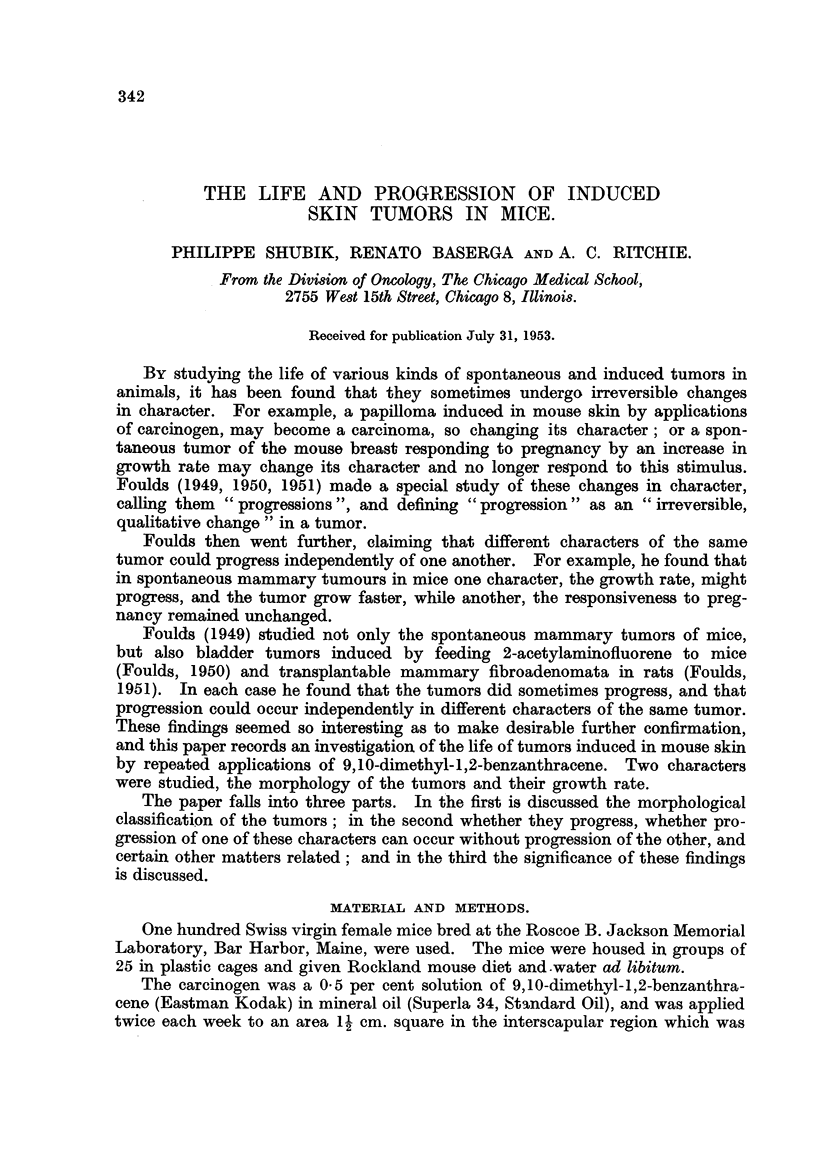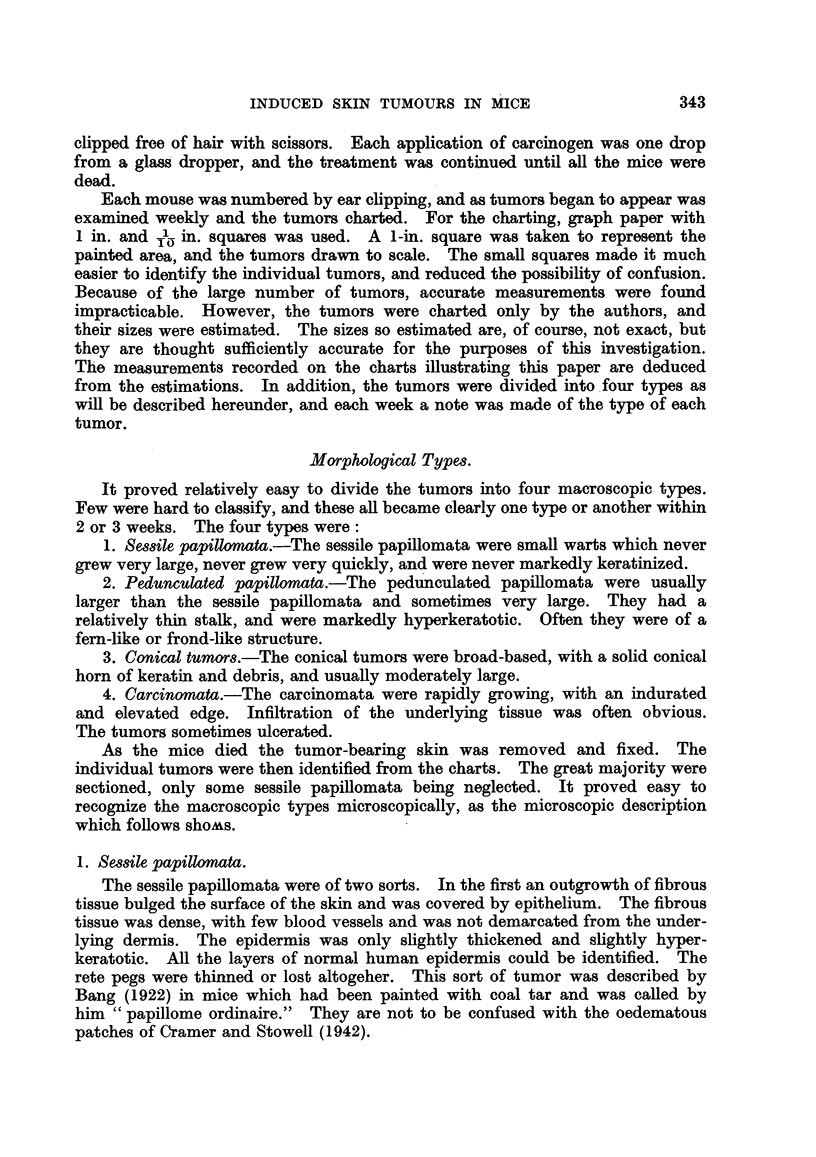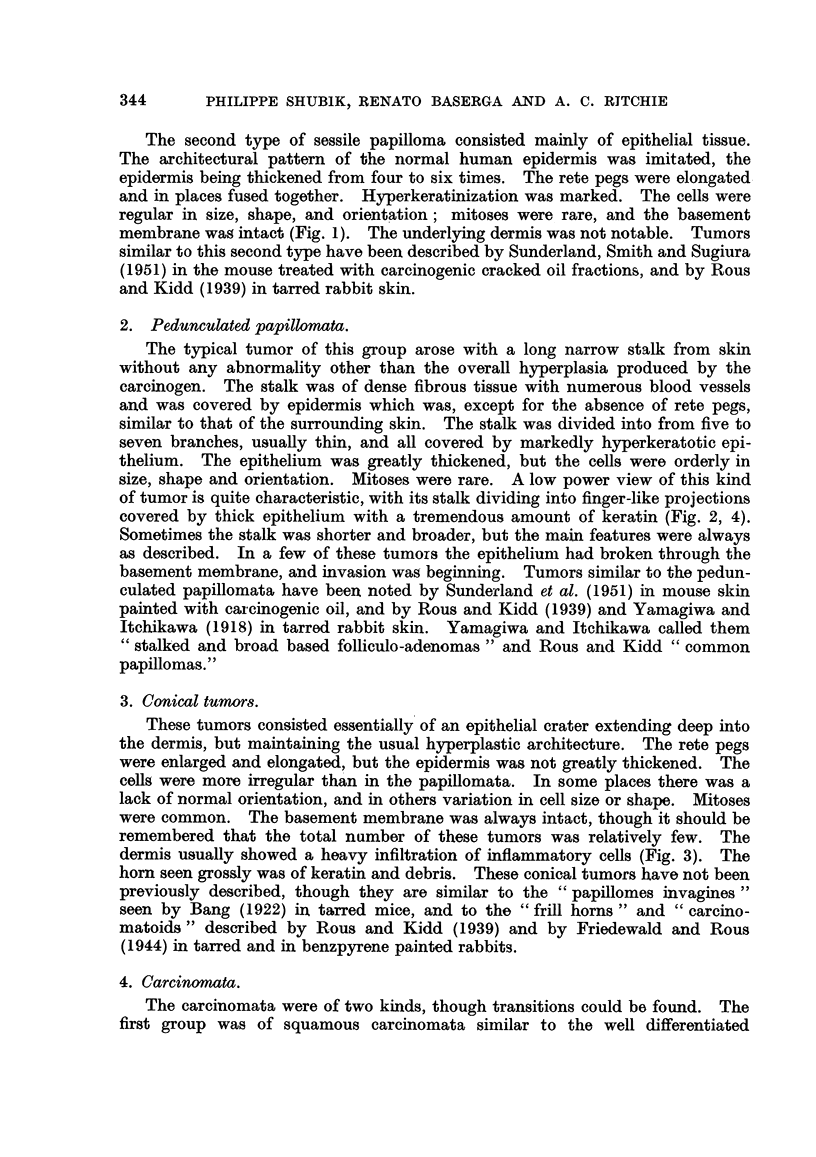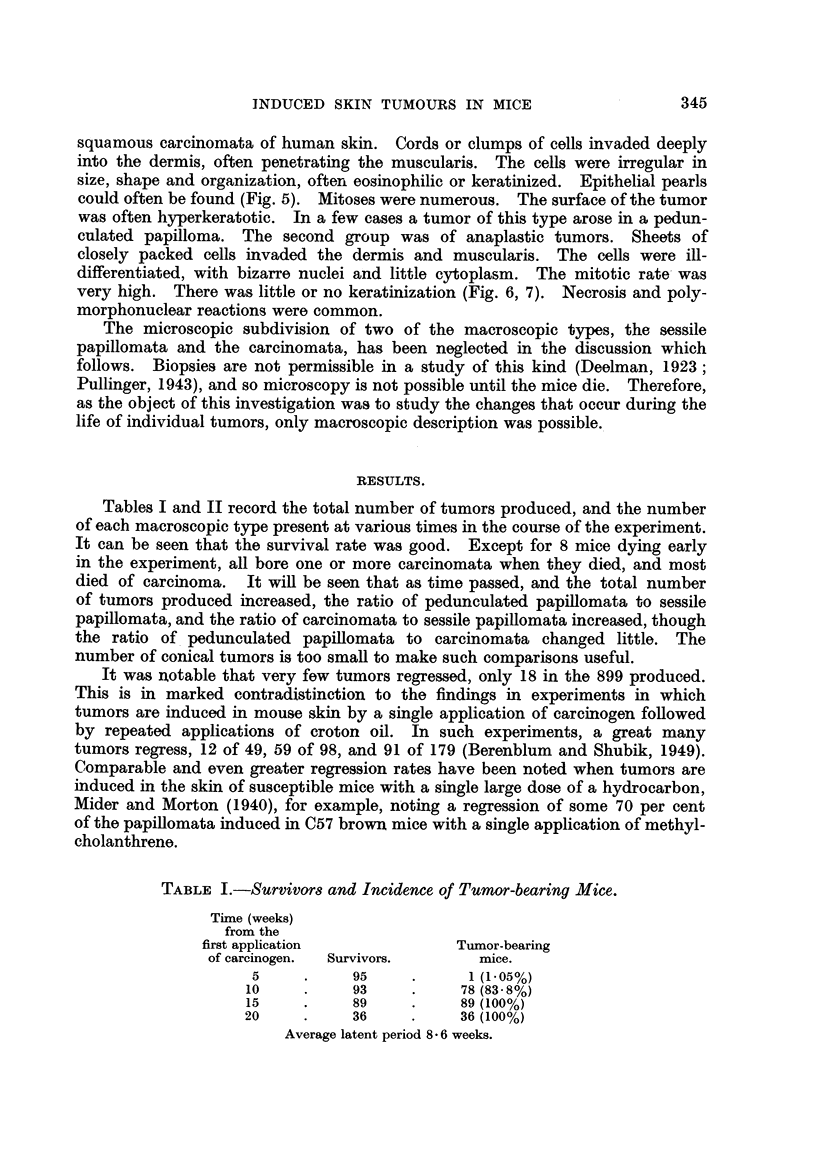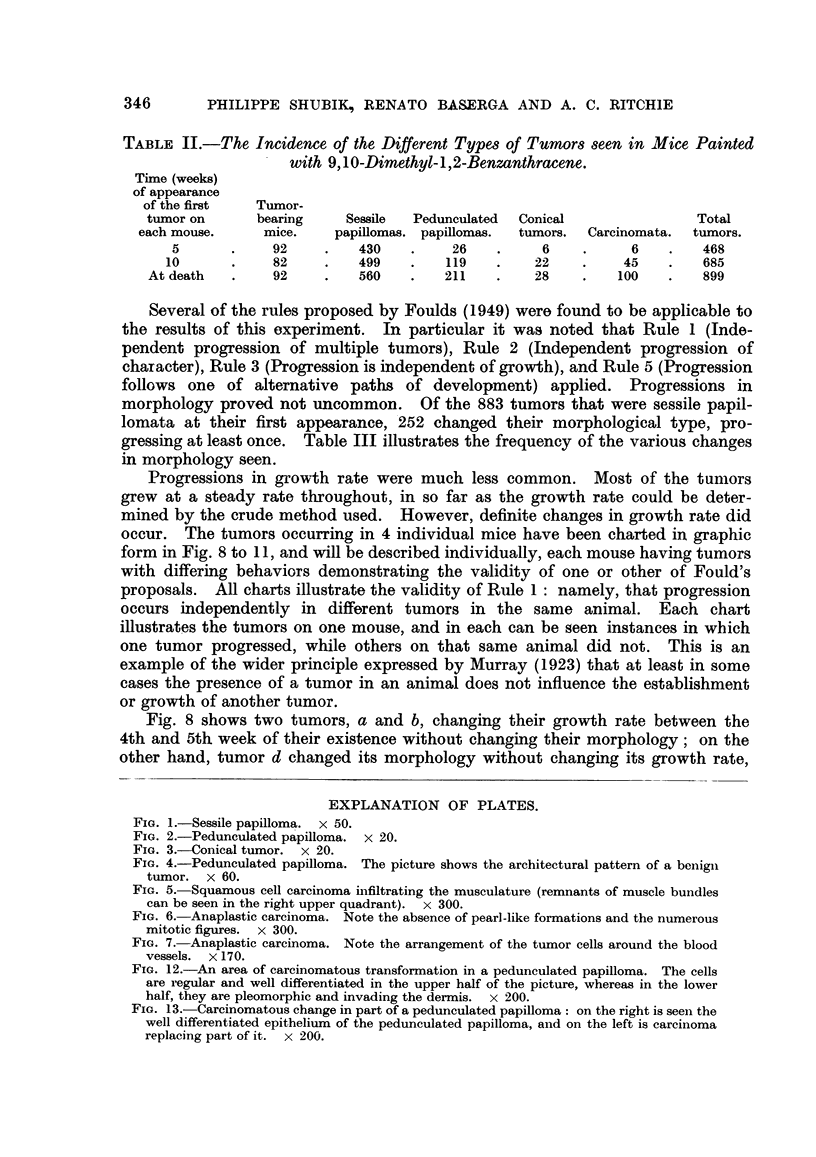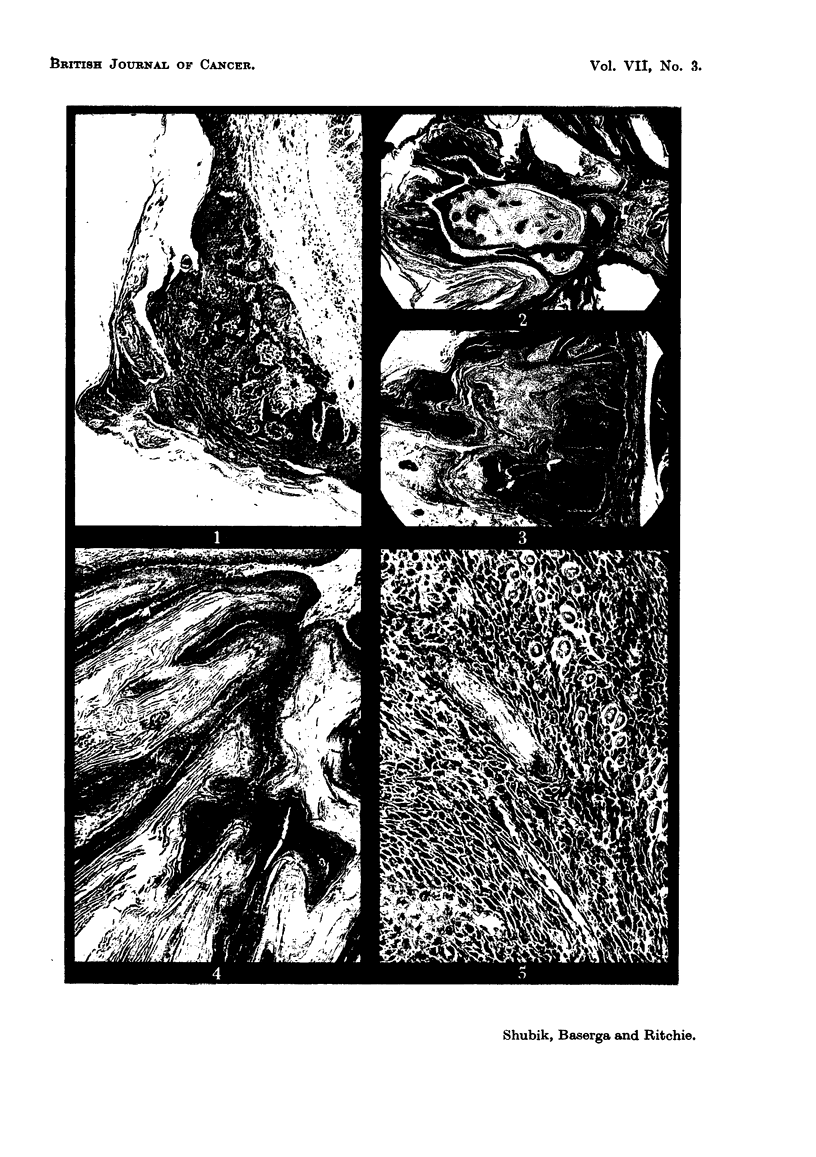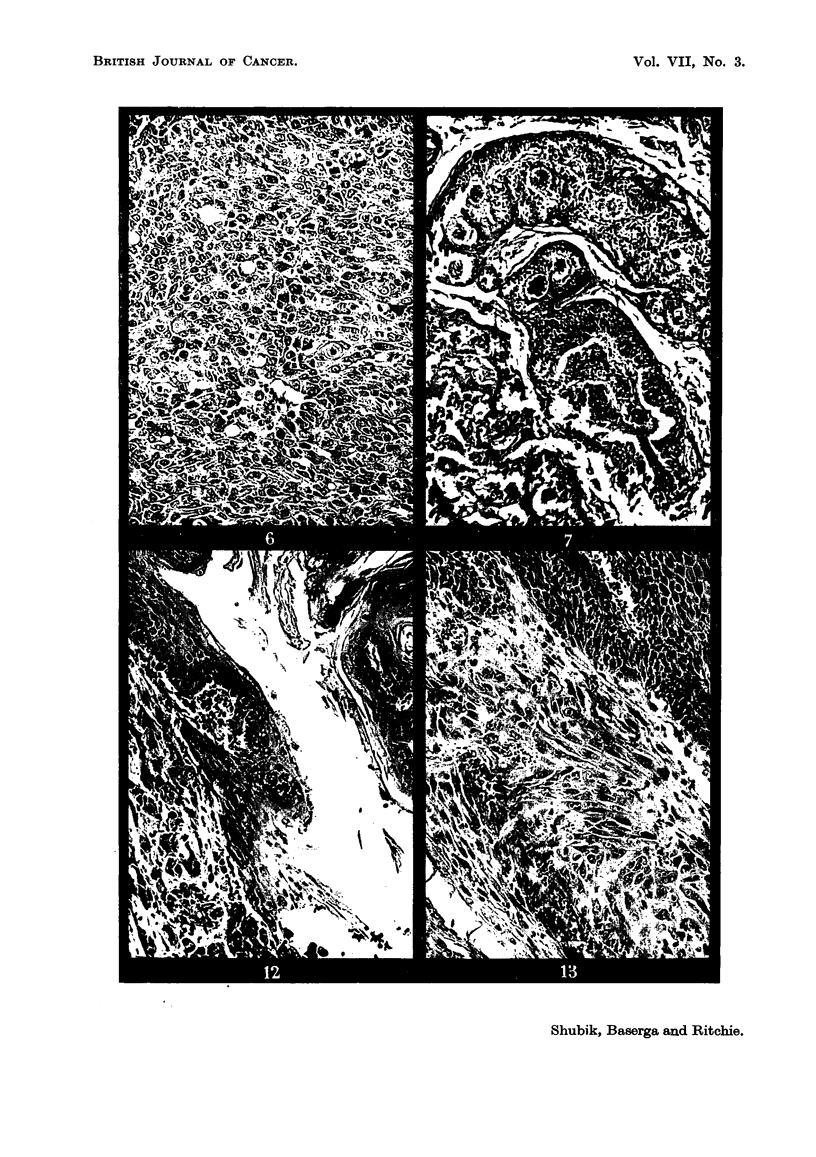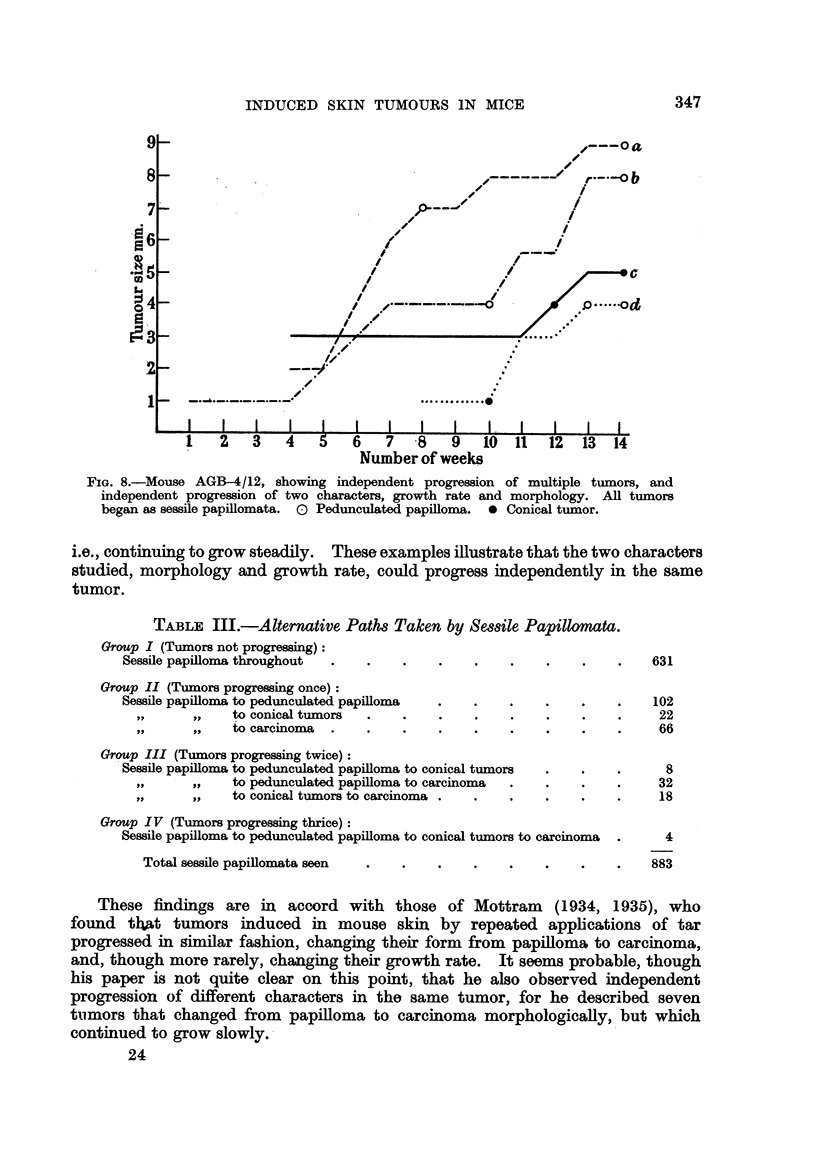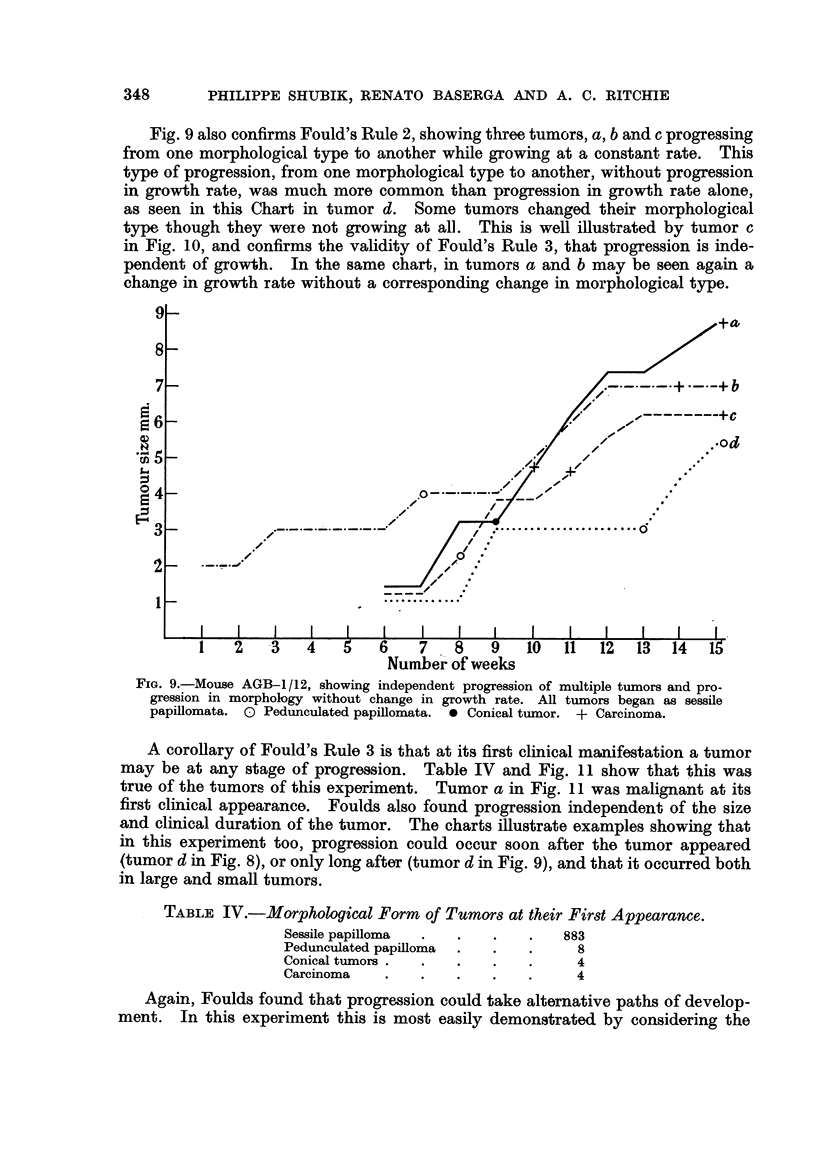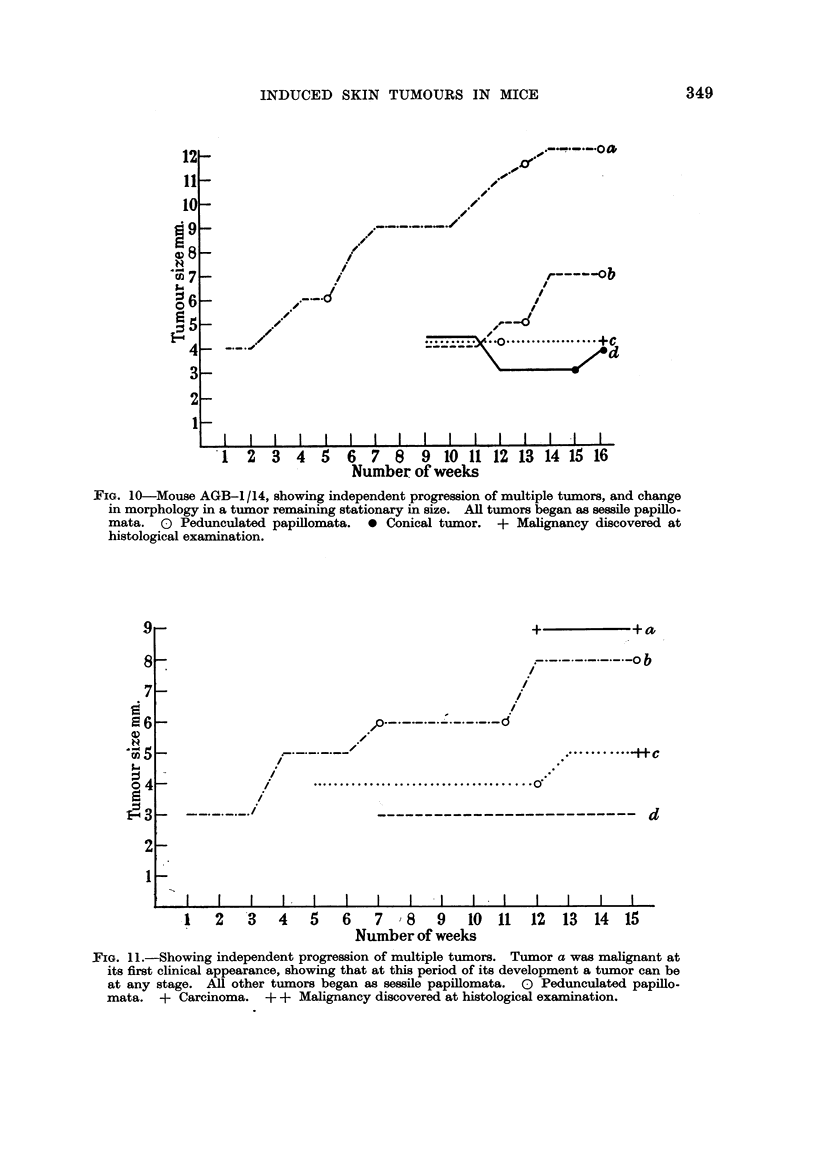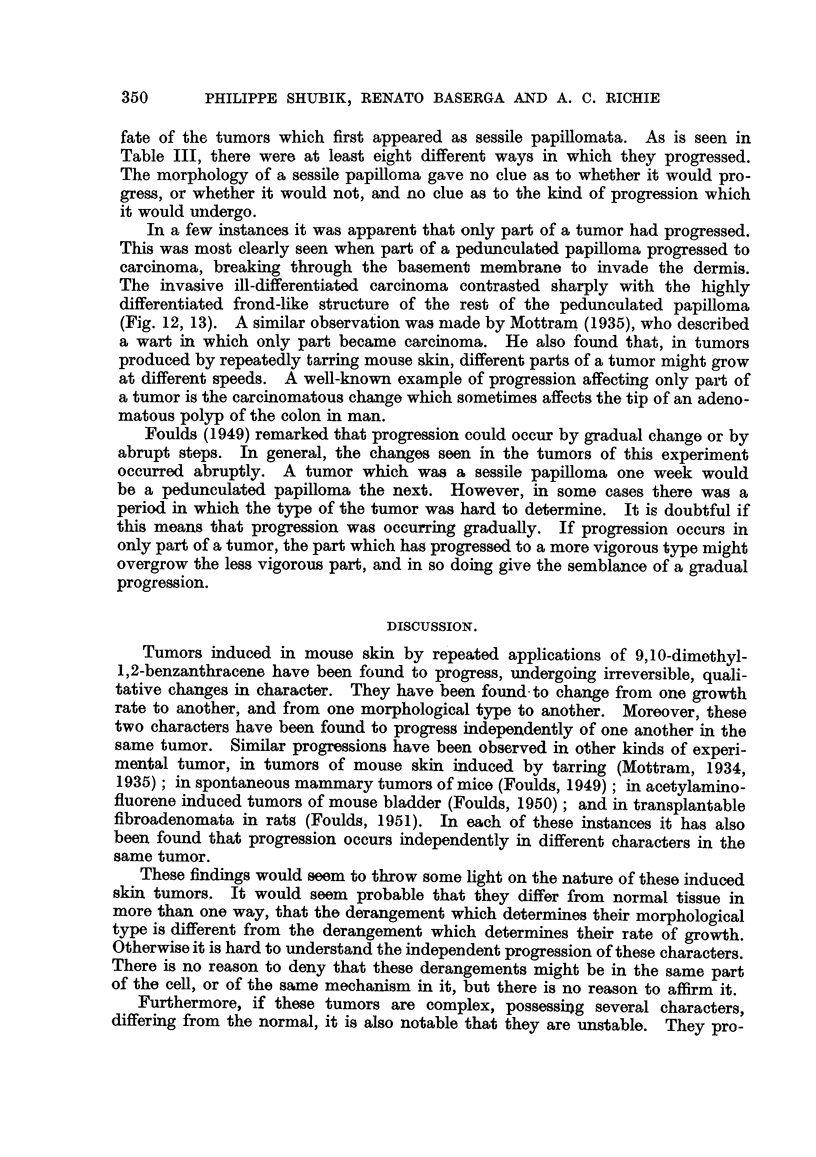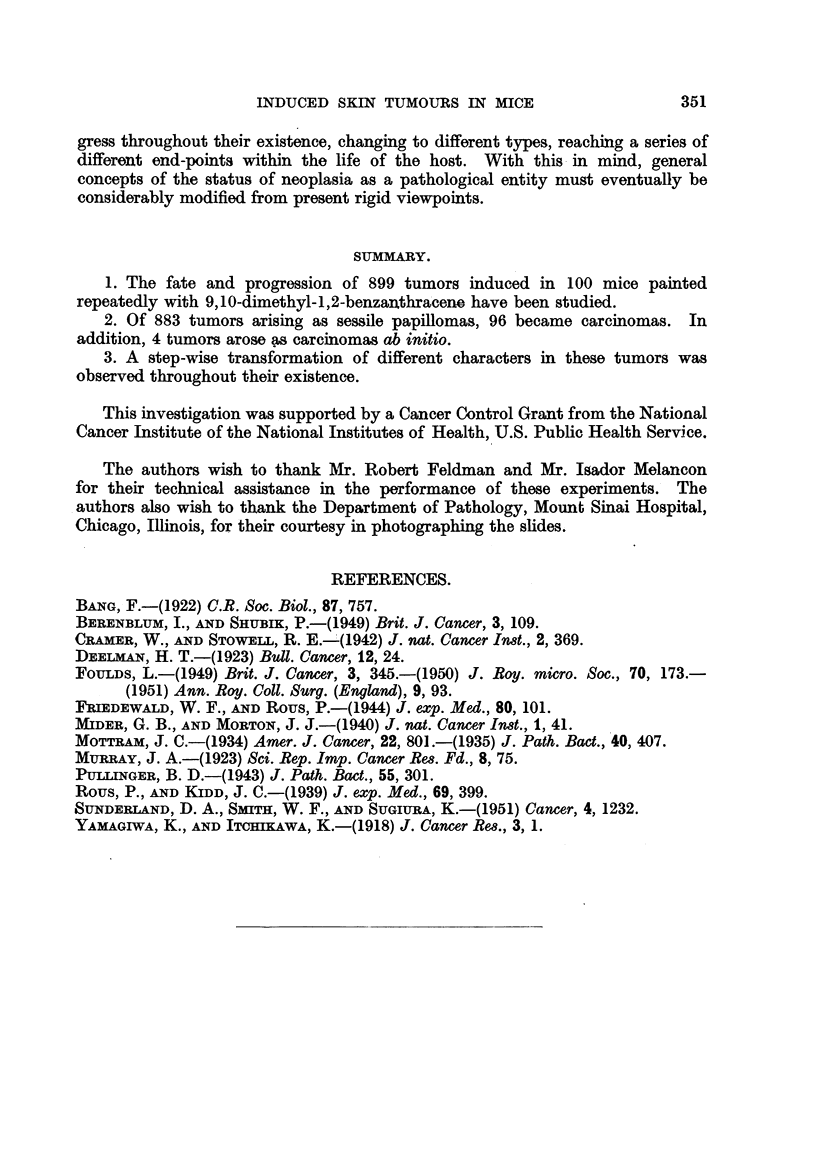# The Life and Progression of Induced Skin Tumors in Mice

**DOI:** 10.1038/bjc.1953.32

**Published:** 1953-09

**Authors:** Philippe Shubik, Renato Baserga, A. C. Ritchie

## Abstract

**Images:**


### THE LIFE AND PROGRESSION OF INDUCED SKIN TUMORS IN MICE.

PHILIPPE SHUBIK, RENATO BASERGA AND A. C. RITCHIE.

*From the Division of Oncology, The Chicago Medical School, 2755 West 15th Street, Chicago 8, Illinois.*

Received for publication July 31, 1953.

BY studying the life of various kinds of spontaneous and induced tumors in animals, it has been found that they sometimes undergo irreversible changes in character. For example, a papilloma induced in mouse skin by applications of carcinogen, may become a carcinoma, so changing its character ; or a spontaneous tumor of the mouse breast responding to pregnancy by an increase in growth rate may change its character and no longer respond to this stimulus. Foulds (1949, 1950, 1951) made a special study of these changes in character, calling them " progressions ", and defining " progression " as an " irreversible, qualitative change " in a tumor.

Foulds then went further, claiming that different characters of the same tumor could progress independently of one another. For example, he found that in spontaneous mammary tumours in mice one character, the growth rate, might progress, and the tumor grow faster, while another, the responsiveness to pregnancy remained unchanged.

Foulds (1949) studied not only the spontaneous mammary tumors of mice, but also bladder tumors induced by feeding 2-acetylaminofluorene to mice (Foulds, 1950) and transplantable mammary fibroadenomata in rats (Foulds, 1951). In each case he found that the tumors did sometimes progress, and that progression could occur independently in different characters of the same tumor. These findings seemed so interesting as to make desirable further confirmation, and this paper records an investigation of the life of tumors induced in mouse skin by repeated applications of 9,10-dimethyl-1,2-benzanthracene. Two characters were studied, the morphology of the tumors and their growth rate.

The paper falls into three parts. In the first is discussed the morphological classification of the tumors ; in the second whether they progress, whether progression of one of these characters can occur without progression of the other, and certain other matters related ; and in the third the significance of these findings is discussed.

### MATERIAL AND METHODS.

One hundred Swiss virgin female mice bred at the Roscoe B. Jackson Memorial Laboratory, Bar Harbor, Maine, were used. The mice were housed in groups of 25 in plastic cages and given Rockland mouse diet and water *ad libitum.*

The carcinogen was a 0·5 per cent solution of 9,10-dimethyl-1,2-benzanthracene (Eastman Kodak) in mineral oil (Superla 34, Standard Oil), and was applied twice each week to an area $1\frac{1}{2}$ cm. square in the interscapular region which was clipped free of hair with scissors. Each application of carcinogen was one drop from a glass dropper, and the treatment was continued until all the mice were dead.

Each mouse was numbered by ear clipping, and as tumors began to appear was examined weekly and the tumors charted. For the charting, graph paper with 1 in. and $\frac{1}{10}$ in. squares was used. A 1-in. square was taken to represent the painted area, and the tumors drawn to scale. The small squares made it much easier to identify the individual tumors, and reduced the possibility of confusion. Because of the large number of tumors, accurate measurements were found impracticable. However, the tumors were charted only by the authors, and their sizes were estimated. The sizes so estimated are, of course, not exact, but they are thought sufficiently accurate for the purposes of this investigation. The measurements recorded on the charts illustrating this paper are deduced from the estimations. In addition, the tumors were divided into four types as will be described hereunder, and each week a note was made of the type of each tumor.

### *Morphological Types.*

It proved relatively easy to divide the tumors into four macroscopic types. Few were hard to classify, and these all became clearly one type or another within 2 or 3 weeks. The four types were :

1. *Sessile papillomata.*—The sessile papillomata were small warts which never grew very large, never grew very quickly, and were never markedly keratinized.

2. *Pedunculated papillomata.*—The pedunculated papillomata were usually larger than the sessile papillomata and sometimes very large. They had a relatively thin stalk, and were markedly hyperkeratotic. Often they were of a fern-like or frond-like structure.

3. *Conical tumors.*—The conical tumors were broad-based, with a solid conical horn of keratin and debris, and usually moderately large.

4. *Carcinomata.*—The carcinomata were rapidly growing, with an indurated and elevated edge. Infiltration of the underlying tissue was often obvious. The tumors sometimes ulcerated.

As the mice died the tumor-bearing skin was removed and fixed. The individual tumors were then identified from the charts. The great majority were sectioned, only some sessile papillomata being neglected. It proved easy to recognize the macroscopic types microscopically, as the microscopic description which follows shows.

### 1. *Sessile papillomata.*

The sessile papillomata were of two sorts. In the first an outgrowth of fibrous tissue bulged the surface of the skin and was covered by epithelium. The fibrous tissue was dense, with few blood vessels and was not demarcated from the underlying dermis. The epidermis was only slightly thickened and slightly hyperkeratotic. All the layers of normal human epidermis could be identified. The rete pegs were thinned or lost altogeher. This sort of tumor was described by Bang (1922) in mice which had been painted with coal tar and was called by him " papillome ordinaire." They are not to be confused with the oedematous patches of Cramer and Stowell (1942).

The second type of sessile papilloma consisted mainly of epithelial tissue. The architectural pattern of the normal human epidermis was imitated, the epidermis being thickened from four to six times. The rete pegs were elongated and in places fused together. Hyperkeratinization was marked. The cells were regular in size, shape, and orientation ; mitoses were rare, and the basement membrane was intact (Fig. 1). The underlying dermis was not notable. Tumors similar to this second type have been described by Sunderland, Smith and Sugiura (1951) in the mouse treated with carcinogenic cracked oil fractions, and by Rous and Kidd (1939) in tarred rabbit skin.

2. *Pedunculated papillomata.*

The typical tumor of this group arose with a long narrow stalk from skin without any abnormality other than the overall hyperplasia produced by the carcinogen. The stalk was of dense fibrous tissue with numerous blood vessels and was covered by epidermis which was, except for the absence of rete pegs, similar to that of the surrounding skin. The stalk was divided into from five to seven branches, usually thin, and all covered by markedly hyperkeratotic epithelium. The epithelium was greatly thickened, but the cells were orderly in size, shape and orientation. Mitoses were rare. A low power view of this kind of tumor is quite characteristic, with its stalk dividing into finger-like projections covered by thick epithelium with a tremendous amount of keratin (Fig. 2, 4). Sometimes the stalk was shorter and broader, but the main features were always as described. In a few of these tumors the epithelium had broken through the basement membrane, and invasion was beginning. Tumors similar to the pedunculated papillomata have been noted by Sunderland *et al.* (1951) in mouse skin painted with carcinogenic oil, and by Rous and Kidd (1939) and Yamagiwa and Itchikawa (1918) in tarred rabbit skin. Yamagiwa and Itchikawa called them " stalked and broad based folliculo-adenomas " and Rous and Kidd " common papillomas."

3. *Conical tumors.*

These tumors consisted essentially of an epithelial crater extending deep into the dermis, but maintaining the usual hyperplastic architecture. The rete pegs were enlarged and elongated, but the epidermis was not greatly thickened. The cells were more irregular than in the papillomata. In some places there was a lack of normal orientation, and in others variation in cell size or shape. Mitoses were common. The basement membrane was always intact, though it should be remembered that the total number of these tumors was relatively few. The dermis usually showed a heavy infiltration of inflammatory cells (Fig. 3). The horn seen grossly was of keratin and debris. These conical tumors have not been previously described, though they are similar to the " papillomes invagines " seen by Bang (1922) in tarred mice, and to the " frill horns " and " carcinomatoids " described by Rous and Kidd (1939) and by Friedewald and Rous (1944) in tarred and in benzpyrene painted rabbits.

4. *Carcinomata.*

The carcinomata were of two kinds, though transitions could be found. The first group was of squamous carcinomata similar to the well differentiated squamous carcinomata of human skin. Cords or clumps of cells invaded deeply into the dermis, often penetrating the muscularis. The cells were irregular in size, shape and organization, often eosinophilic or keratinized. Epithelial pearls could often be found (Fig. 5). Mitoses were numerous. The surface of the tumor was often hyperkeratotic. In a few cases a tumor of this type arose in a pedunculated papilloma. The second group was of anaplastic tumors. Sheets of closely packed cells invaded the dermis and muscularis. The cells were ill-differentiated, with bizarre nuclei and little cytoplasm. The mitotic rate was very high. There was little or no keratinization (Fig. 6, 7). Necrosis and polymorphonuclear reactions were common.

The microscopic subdivision of two of the macroscopic types, the sessile papillomata and the carcinomata, has been neglected in the discussion which follows. Biopsies are not permissible in a study of this kind (Deelman, 1923; Pullinger, 1943), and so microscopy is not possible until the mice die. Therefore, as the object of this investigation was to study the changes that occur during the life of individual tumors, only macroscopic description was possible.

### RESULTS.

Tables I and II record the total number of tumors produced, and the number of each macroscopic type present at various times in the course of the experiment. It can be seen that the survival rate was good. Except for 8 mice dying early in the experiment, all bore one or more carcinomata when they died, and most died of carcinoma. It will be seen that as time passed, and the total number of tumors produced increased, the ratio of pedunculated papillomata to sessile papillomata, and the ratio of carcinomata to sessile papillomata increased, though the ratio of pedunculated papillomata to carcinomata changed little. The number of conical tumors is too small to make such comparisons useful.

It was notable that very few tumors regressed, only 18 in the 899 produced. This is in marked contradistinction to the findings in experiments in which tumors are induced in mouse skin by a single application of carcinogen followed by repeated applications of croton oil. In such experiments, a great many tumors regress, 12 of 49, 59 of 98, and 91 of 179 (Berenblum and Shubik, 1949). Comparable and even greater regression rates have been noted when tumors are induced in the skin of susceptible mice with a single large dose of a hydrocarbon, Mider and Morton (1940), for example, noting a regression of some 70 per cent of the papillomata induced in C57 brown mice with a single application of methylcholanthrene.

TABLE I.—*Survivors and Incidence of Tumor-bearing Mice.*

| Time (weeks) from the first application of carcinogen. | Survivors. | Tumor-bearing mice. |
|---|---|---|
| 5 | 95 | 1 (1·05%) |
| 10 | 93 | 78 (83·8%) |
| 15 | 89 | 89 (100%) |
| 20 | 36 | 36 (100%) |

Average latent period 8·6 weeks.

TABLE II.—*The Incidence of the Different Types of Tumors seen in Mice Painted with* 9,10-*Dimethyl*-1,2-*Benzanthracene.*

| Time (weeks) of appearance of the first tumor on each mouse. | Tumor-bearing mice. | Sessile papillomas. | Pedunculated papillomas. | Conical tumors. | Carcinomata. | Total tumors. |
|---|---|---|---|---|---|---|
| 5 | 92 | 430 | 26 | 6 | 6 | 468 |
| 10 | 82 | 499 | 119 | 22 | 45 | 685 |
| At death | 92 | 560 | 211 | 28 | 100 | 899 |

Several of the rules proposed by Foulds (1949) were found to be applicable to the results of this experiment. In particular it was noted that Rule 1 (Independent progression of multiple tumors), Rule 2 (Independent progression of character), Rule 3 (Progression is independent of growth), and Rule 5 (Progression follows one of alternative paths of development) applied. Progressions in morphology proved not uncommon. Of the 883 tumors that were sessile papillomata at their first appearance, 252 changed their morphological type, progressing at least once. Table III illustrates the frequency of the various changes in morphology seen.

Progressions in growth rate were much less common. Most of the tumors grew at a steady rate throughout, in so far as the growth rate could be determined by the crude method used. However, definite changes in growth rate did occur. The tumors occurring in 4 individual mice have been charted in graphic form in Fig. 8 to 11, and will be described individually, each mouse having tumors with differing behaviors demonstrating the validity of one or other of Fould's proposals. All charts illustrate the validity of Rule 1 : namely, that progression occurs independently in different tumors in the same animal. Each chart illustrates the tumors on one mouse, and in each can be seen instances in which one tumor progressed, while others on that same animal did not. This is an example of the wider principle expressed by Murray (1923) that at least in some cases the presence of a tumor in an animal does not influence the establishment or growth of another tumor.

Fig. 8 shows two tumors, *a* and *b*, changing their growth rate between the 4th and 5th week of their existence without changing their morphology ; on the other hand, tumor *d* changed its morphology without changing its growth rate,

---

EXPLANATION OF PLATES.

FIG. 1.—Sessile papilloma. × 50.
FIG. 2.—Pedunculated papilloma. × 20.
FIG. 3.—Conical tumor. × 20.
FIG. 4.—Pedunculated papilloma. The picture shows the architectural pattern of a benign tumor. × 60.
FIG. 5.—Squamous cell carcinoma infiltrating the musculature (remnants of muscle bundles can be seen in the right upper quadrant). × 300.
FIG. 6.—Anaplastic carcinoma. Note the absence of pearl-like formations and the numerous mitotic figures. × 300.
FIG. 7.—Anaplastic carcinoma. Note the arrangement of the tumor cells around the blood vessels. ×170.
FIG. 12.—An area of carcinomatous transformation in a pedunculated papilloma. The cells are regular and well differentiated in the upper half of the picture, whereas in the lower half, they are pleomorphic and invading the dermis. × 200.
FIG. 13.—Carcinomatous change in part of a pedunculated papilloma : on the right is seen the well differentiated epithelium of the pedunculated papilloma, and on the left is carcinoma replacing part of it. × 200.

1
2
3
4
5

Shubik, Baserga and Ritchie.

FIG. 8.—Mouse AGB–4/12, showing independent progression of multiple tumors, and independent progression of two characters, growth rate and morphology. All tumors began as sessile papillomata. ⊙ Pedunculated papilloma. ● Conical tumor.

i.e., continuing to grow steadily. These examples illustrate that the two characters studied, morphology and growth rate, could progress independently in the same tumor.

TABLE III.—*Alternative Paths Taken by Sessile Papillomata.*

| | |
|---|---|
| *Group I* (Tumors not progressing): | |
| Sessile papilloma throughout | 631 |
| *Group II* (Tumors progressing once): | |
| Sessile papilloma to pedunculated papilloma | 102 |
| ,, ,, to conical tumors | 22 |
| ,, ,, to carcinoma | 66 |
| *Group III* (Tumors progressing twice): | |
| Sessile papilloma to pedunculated papilloma to conical tumors | 8 |
| ,, ,, to pedunculated papilloma to carcinoma | 32 |
| ,, ,, to conical tumors to carcinoma | 18 |
| *Group IV* (Tumors progressing thrice): | |
| Sessile papilloma to pedunculated papilloma to conical tumors to carcinoma | 4 |
| Total sessile papillomata seen | 883 |

These findings are in accord with those of Mottram (1934, 1935), who found that tumors induced in mouse skin by repeated applications of tar progressed in similar fashion, changing their form from papilloma to carcinoma, and, though more rarely, changing their growth rate. It seems probable, though his paper is not quite clear on this point, that he also observed independent progression of different characters in the same tumor, for he described seven tumors that changed from papilloma to carcinoma morphologically, but which continued to grow slowly.

Fig. 9 also confirms Fould's Rule 2, showing three tumors, *a*, *b* and *c* progressing from one morphological type to another while growing at a constant rate. This type of progression, from one morphological type to another, without progression in growth rate, was much more common than progression in growth rate alone, as seen in this Chart in tumor *d*. Some tumors changed their morphological type though they were not growing at all. This is well illustrated by tumor *c* in Fig. 10, and confirms the validity of Fould's Rule 3, that progression is independent of growth. In the same chart, in tumors *a* and *b* may be seen again a change in growth rate without a corresponding change in morphological type.

Fig. 9.—Mouse AGB–1/12, showing independent progression of multiple tumors and progression in morphology without change in growth rate. All tumors began as sessile papillomata. ○ Pedunculated papillomata. ● Conical tumor. + Carcinoma.

A corollary of Fould's Rule 3 is that at its first clinical manifestation a tumor may be at any stage of progression. Table IV and Fig. 11 show that this was true of the tumors of this experiment. Tumor *a* in Fig. 11 was malignant at its first clinical appearance. Foulds also found progression independent of the size and clinical duration of the tumor. The charts illustrate examples showing that in this experiment too, progression could occur soon after the tumor appeared (tumor *d* in Fig. 8), or only long after (tumor *d* in Fig. 9), and that it occurred both in large and small tumors.

Table IV.—*Morphological Form of Tumors at their First Appearance.*

| | |
|---|---|
| Sessile papilloma . . . . | 883 |
| Pedunculated papilloma . . . | 8 |
| Conical tumors . . . . . | 4 |
| Carcinoma . . . . . | 4 |

Again, Foulds found that progression could take alternative paths of development. In this experiment this is most easily demonstrated by considering the

FIG. 10—Mouse AGB–1/14, showing independent progression of multiple tumors, and change in morphology in a tumor remaining stationary in size. All tumors began as sessile papillomata. ⊙ Pedunculated papillomata. ● Conical tumor. + Malignancy discovered at histological examination.

FIG. 11.—Showing independent progression of multiple tumors. Tumor *a* was malignant at its first clinical appearance, showing that at this period of its development a tumor can be at any stage. All other tumors began as sessile papillomata. ⊙ Pedunculated papillomata. + Carcinoma. ++ Malignancy discovered at histological examination.

fate of the tumors which first appeared as sessile papillomata. As is seen in Table III, there were at least eight different ways in which they progressed. The morphology of a sessile papilloma gave no clue as to whether it would progress, or whether it would not, and no clue as to the kind of progression which it would undergo.

In a few instances it was apparent that only part of a tumor had progressed. This was most clearly seen when part of a pedunculated papilloma progressed to carcinoma, breaking through the basement membrane to invade the dermis. The invasive ill-differentiated carcinoma contrasted sharply with the highly differentiated frond-like structure of the rest of the pedunculated papilloma (Fig. 12, 13). A similar observation was made by Mottram (1935), who described a wart in which only part became carcinoma. He also found that, in tumors produced by repeatedly tarring mouse skin, different parts of a tumor might grow at different speeds. A well-known example of progression affecting only part of a tumor is the carcinomatous change which sometimes affects the tip of an adenomatous polyp of the colon in man.

Foulds (1949) remarked that progression could occur by gradual change or by abrupt steps. In general, the changes seen in the tumors of this experiment occurred abruptly. A tumor which was a sessile papilloma one week would be a pedunculated papilloma the next. However, in some cases there was a period in which the type of the tumor was hard to determine. It is doubtful if this means that progression was occurring gradually. If progression occurs in only part of a tumor, the part which has progressed to a more vigorous type might overgrow the less vigorous part, and in so doing give the semblance of a gradual progression.

### DISCUSSION.

Tumors induced in mouse skin by repeated applications of 9,10-dimethyl-1,2-benzanthracene have been found to progress, undergoing irreversible, qualitative changes in character. They have been found to change from one growth rate to another, and from one morphological type to another. Moreover, these two characters have been found to progress independently of one another in the same tumor. Similar progressions have been observed in other kinds of experimental tumor, in tumors of mouse skin induced by tarring (Mottram, 1934, 1935) ; in spontaneous mammary tumors of mice (Foulds, 1949) ; in acetylaminofluorene induced tumors of mouse bladder (Foulds, 1950) ; and in transplantable fibroadenomata in rats (Foulds, 1951). In each of these instances it has also been found that progression occurs independently in different characters in the same tumor.

These findings would seem to throw some light on the nature of these induced skin tumors. It would seem probable that they differ from normal tissue in more than one way, that the derangement which determines their morphological type is different from the derangement which determines their rate of growth. Otherwise it is hard to understand the independent progression of these characters. There is no reason to deny that these derangements might be in the same part of the cell, or of the same mechanism in it, but there is no reason to affirm it.

Furthermore, if these tumors are complex, possessing several characters, differing from the normal, it is also notable that they are unstable. They progress throughout their existence, changing to different types, reaching a series of different end-points within the life of the host. With this in mind, general concepts of the status of neoplasia as a pathological entity must eventually be considerably modified from present rigid viewpoints.

### SUMMARY.

1. The fate and progression of 899 tumors induced in 100 mice painted repeatedly with 9,10-dimethyl-1,2-benzanthracene have been studied.

2. Of 883 tumors arising as sessile papillomas, 96 became carcinomas. In addition, 4 tumors arose as carcinomas *ab initio*.

3. A step-wise transformation of different characters in these tumors was observed throughout their existence.

This investigation was supported by a Cancer Control Grant from the National Cancer Institute of the National Institutes of Health, U.S. Public Health Service.

The authors wish to thank Mr. Robert Feldman and Mr. Isador Melancon for their technical assistance in the performance of these experiments. The authors also wish to thank the Department of Pathology, Mount Sinai Hospital, Chicago, Illinois, for their courtesy in photographing the slides.

### REFERENCES.

BANG, F.—(1922) *C.R. Soc. Biol.*, **87**, 757.
BERENBLUM, I., AND SHUBIK, P.—(1949) *Brit. J. Cancer*, **3**, 109.
CRAMER, W., AND STOWELL, R. E.—(1942) *J. nat. Cancer Inst.*, **2**, 369.
DEELMAN, H. T.—(1923) *Bull. Cancer*, **12**, 24.
FOULDS, L.—(1949) *Brit. J. Cancer*, **3**, 345.—(1950) *J. Roy. micro. Soc.*, **70**, 173.—(1951) *Ann. Roy. Coll. Surg.* (*England*), **9**, 93.
FRIEDEWALD, W. F., AND ROUS, P.—(1944) *J. exp. Med.*, **80**, 101.
MIDER, G. B., AND MORTON, J. J.—(1940) *J. nat. Cancer Inst.*, **1**, 41.
MOTTRAM, J. C.—(1934) *Amer. J. Cancer*, **22**, 801.—(1935) *J. Path. Bact.*, **40**, 407.
MURRAY, J. A.—(1923) *Sci. Rep. Imp. Cancer Res. Fd.*, **8**, 75.
PULLINGER, B. D.—(1943) *J. Path. Bact.*, **55**, 301.
ROUS, P., AND KIDD, J. C.—(1939) *J. exp. Med.*, **69**, 399.
SUNDERLAND, D. A., SMITH, W. F., AND SUGIURA, K.—(1951) *Cancer*, **4**, 1232.
YAMAGIWA, K., AND ITCHIKAWA, K.—(1918) *J. Cancer Res.*, **3**, 1.